# A Novel Approach to Optimize Hot Melt Impregnation in Terms of Amorphization Efficiency

**DOI:** 10.3390/ijms21114032

**Published:** 2020-06-04

**Authors:** Kamil Garbera, Krzesimir Ciura, Wiesław Sawicki

**Affiliations:** 1 Formulation Department, Tarchomin Pharmaceutical Works “Polfa” S.A., Fleminga 2, 03-176 Warszawa, Poland; kamil.garbera@gmail.com; 2 Department of Physical Chemistry, Medical University of Gdańsk, Hallera 107, 80-416 Gdańsk, Poland; krzesimir.ciura@gumed.edu.pl

**Keywords:** hot melt impregnation, design of experiments, amorphization, ibuprofen

## Abstract

In this study, an innovative methodology to optimize amorphization during the hot melt impregnation (HMI) process was proposed. The novelty of this report revolves around the use of thermal analysis in combination with design of experiments (DoEs) to reduce residual crystallinity during the HMI process. As a model formulation, a mixture of ibuprofen (IBU) and Neusilin was used. The main aim of the study was to identify the critical process parameters of HMI and determine their optimal values to assure a robust impregnation process and possibly the highest possible amorphization rate of IBU. In order to realize this, a DoE approach was proposed based on a face-centered composite design involving three factors. The IBU/Neusilin ratio, the feeding rate, and the screw speed were considered as variables, while the residual crystallinity level of IBU, determined using differential scanning calorimetry (DSC), was measured as the response. Additionally, the stability of IBU under HMI was analyzed using high-performance liquid chromatography to estimate the extent of potential degradation. In order to verify the correctness of the DoE model, tested extrudates were manufactured by HMI and the obtained extrudates were thoroughly examined using scanning electron micrography, X-ray powder diffraction, and DSC.

## 1. Introduction

Hot melt extrusion (HME) is a continuous process originating from the plastic and food industries. At the end of the last century, HME was successfully implemented into pharmaceutical technologies and applied as a production process of various drug delivery systems, such as granules, pellets, and sustained release tablets [1,2]. 

The main idea of HME is to disperse or dissolve an active pharmaceutical ingredient (API) within a polymeric matrix to form a solid dispersion in either a crystalline or amorphous phase, or ideally to create a one-phase solid solution system. This approach is especially useful to overcome problems with bioavailability commonly seen in poorly water-soluble drugs found in oral formulations, since the use of an amorphous form of an active substance is well known to improve oral bioavailability [3]. Amorphous systems possess the highest possible energy levels of all solid-state forms, thereby offering higher solubility, faster dissolution rates, and subsequent potential improvement in bioavailability. However, thermodynamic stability is always a challenge for amorphous substances and presents a main disadvantage of amorphous state exploitation. Physical instability leads to conversion into a crystalline state [4], but physical stability can be significantly increased after HME and formation of an amorphous solid dispersion. 

In addition to standard polymeric substances, applications using inorganic excipients as drug carriers to increase dissolution rates of poorly water-soluble drugs were recently reported and defined as hot melt impregnation (HMI) [5]. HMI is a process that involves applying heat and mechanically created stress to a mixture of processed raw materials and forcing it through a barrel in a powder form. During this process, raw material is transported through the extruder’s barrel by co-rotating twin screws. Heat is applied to the barrel to increase the temperature of the raw material, causing melting of the substances with low melting points. The melted substances penetrate into the open inorganic excipient’s pores; the capillary effect is considered to be a driving force of this phenomenon. As a product of this process, a fine free-flowing extrudate is obtained. Due to different physical forms of the extrudate, the process was named HMI instead of HME [5]. It is worth emphasizing that HMI is quite challenging, due to many factors influencing its performance. 

Currently, several technological design of experiment (DoE) processes are used for optimization [6]. Such approaches maximize knowledge of the process while minimizing the use of resources. DoE is widely used to understand the effects of multidimensional input factors and the interactions between them. The most common way of analyzing obtained data from design of experiments is via the response surface methodology (RMS), where the objective is optimization to find the best set of factor levels to achieve a predefined goal [7].

In this study, an innovative approach to optimize amorphization during the HMI process was proposed. The novelty of this report revolves around the use of thermal analysis in combination with DoE to reduce residual crystallinity during the HMI process. A model formulation mixture of ibuprofen (IBU) and magnesium aluminometasilicate (Neusilin) was used. Ibuprofen’s low melting temperature and Neusilin’s enormous specific surface area created a mixture with favorable physicochemical properties to investigate the process.

The main aim of the study was to identify the critical process parameters of HMI and determine their optimal values, thereby assuring a robust impregnation process and possibly the highest possible amorphization rate of IBU. In order to realize this, a DoE approach was proposed based on a central composite design composed of a fractional factorial and star points placed on the faces of the sides. The IBU/Neusilin ratio, the feeding rate, and the screw speed were considered as the variables, while the residual crystallinity level of IBU, as determined using differential scanning calorimetry (DSC), was measured as the response. To the best of our knowledge, this is the first successful attempt to optimize hot melt impregnation in terms of amorphization efficiency using DSC to determine process efficiency. Additionally, the stability of IBU under HMI was analyzed using high-performance liquid chromatography (HPLC). The correctness of the obtained DoE model was verified, the tested extrudates were manufactured by HMI, and the obtained extrudates were thoroughly examined using scanning electron micrography (SEM), X-ray powder diffraction (XRPD), and differential scanning calorimetry (DSC).

## 2. Results and Discussion

Several studies indicated that HME using polymeric matrices was effective when preparing extrudates of amorphous solid dispersions [8,9,10]. Contrary to organic polymers, only a few studies focused on the applications of inorganic excipients [11,12,13]. Actual reports presented by D. Liynova et al. describe the molecular level insight into hot melt drug loading into mesoporous silica carriers. Authors describe the dissolution rate enhancement of melt loaded IBU, however, loading took place by simple heating of the API and carrier in an electric furnace [14]. Another example of the HME process for drug loading into a mesoporous material is presented by N. Genina et al. Two model drugs that have been presented are IBU and carvedilol. Both APIs were incorporated into the porous carrier together with a Soluplus^®^ copolymer, due to high friction during the process. Authors proved the possibility of preparation of a stable amorphous solid dispersion by HME [15]. The given study demonstrates a feasible technological process of the hot melt impregnation process without application of any polymeric compounds. Another advantage of HMI is the solvent-free process, which was stressed by the Štěpánek group [16]. Furthermore, studies concerning the influences of the essential process parameters of HMI are very limited [17]. Developing an approach to allow optimization and performance evaluation of HMI amorphization in a simple way could contribute to a better understanding of how to use inorganic excipients to stabilize a solid dispersion of an amorphous phase. 

During the preliminary study, the selection of a mesoporous material was conducted. Three different materials were examined, Neusilin US2 (magnesium aluminosilicate), Florite PS-200 (calcium silicate), and Syloid XDP 3150 (silicon dioxide). The introduced carriers varied in many aspects. A comparison of each material property has been summarized in Table S1, moreover, Figure S1 presents SEM images of the characterized materials. All proposed carriers have an enormous specific surface area, porous structure, and very good oil absorption capacity, these parameters were crucial for the hot melt impregnation process material selection. 

To verify the usefulness of the selected materials, an impregnation process was conducted. Each material was blended with ibuprofen in a 1:1 mass ratio. Subsequent blends were transferred to the impregnation process. Process parameters were kept constant for each blend (feeding rate 100g/h, screw speed 20 RPM, L/D ratio 20, temperature profile 70/110/170/130 °C, and cooling rate). The material after the process was analyzed using XRPD. Neusilin US2 provided a smooth amorphous diffractogram without any traces of characteristic Bragg’s peaks, which was shown in Figure S2. For this reason, this material was transferred for further investigation. 

Chemically, Neusilin is a magnesium aluminometasilicate containing tetrahedral or octahedral Al, octahedral Mg, and tetrahedral Si [18], forming a random complex of three-dimensional amorphous structures. Similarly to other complex silicates, various types of silanol groups can be observed on the surface of Neusilin, consequently making hydrogen bond interactions possible [18]. It is worth emphasizing that, as a matrix substance, Neusilin has an enormous specific surface area (around 300 m^2^/g) and a high percentage of open pores. Physically, Neusilin has a sphere-like structure, with a mean particle size of 100 µm. These physical parameters provide favorable mechanical properties. Neusilin shows an outstanding flowability and mixability with other substances and is highly compressible, allowing high hardness to be obtained at low compression forces. Several studies indicated that Neusilin improved the formation and stabilization of amorphous forms of various APIs, such as ketoprofen, indomethacin, naproxen, and progesterone [11,18]. Moreover, Neusilin is a commercially available, cost-effective, mesoporous excipient, which is generally a significant limitation of mesoporous silica materials. Previous research indicated that mesoporous materials significantly improved in vitro solubility as well as the bioavailability of poorly soluble drugs [19,20,21]. However, mesoporous silica is still not available in bulk quantities and at a reasonable price. The current protocols for its synthesis include the use of surfactants as pore-forming templates, which make this process expensive and unfavorable for the environment [21,22]. In connection to the above, it should be stressed that at present, Neusilin is superior to other mesoporous silica materials with regard to the available industrial technology. Among various types of Nesilin (US2, UFL2, S1, and S2), Neusilin US2 has been selected based on its physicochemical properties. Other grades of Neusilin do not offer all the benefits such as good compressibility, superior flowability, enormous specific surface area, neutral pH, and appropriate particle size distribution. All these aspects make Neusilin US2 more desirable for use over other grades. The Neusilin UFL2 has a small particle size and thus poor flowability. Grades S1 and S2 have three-times smaller specific surface areas, which could make impregnation difficult. Neusilin US2 was the ideal candidate for this application.

IBU is a poorly water-soluble drug, characterized by dissolution-limited oral bioavailability and high permeability (BCS II). For these reasons, many research groups are interested in establishing solid dosage forms of IBU with improved solubility and bioavailability. Although, the use of solid dispersions (SDs) has been reported as an effective tool to enhance IBU solubility [23,24], the large quantity of polymer increases the bulk of the final dosage form, which limits the use of these formulations at the industrial scale [25]. The toxicity aspect was recognized as a limitation of ionic liquids [26] and self-assembled mixed micelles [27] were applied to improve its solubility. IBU was chosen as the model substance due to its specific physicochemical properties, including its needle-like particle shape, which results in poor flowability and a low melting point, thereby causing problems with sticking during tablet compression; these issues are solved by HMI. In the literature reports presented, the mesoporous magnesium carbonate (Upsalite) [21] and mesostructured silica (MCM-41) [28] formulations of amorphous IBU have been presented. IBU penetrates Neusilin’s pores and alters its shape according to the pore size, which greatly improves the flowability. At the same time, IBU is not exposed to the heat created by the friction between the tablet press tool and the compressed material. On the other hand, the HMI process enables transformation from the crystalline form to the amorphous phase since the narrow pores in mesoporous materials prevent the crystallization of loaded IBU molecules [28]. Another advantage of choosing IBU–Neusilin mixtures is that the interaction between IBU and Neusilin prevents the evaporation of IBU at high temperatures, which is highly desirable in technologies that involve heating [18]. 

To transform IBU from a crystalline into an amorphous form, the HMI process was introduced using an adapted method proposed by Maniruzzaman et al., who used inorganic excipients as drug carriers to manufacture solid dispersions of indomethacin [29]. In our previous study, we reported a successful amorphization of IBU by HMI based on this protocol [5]. Considering the stress conditions under which HMI is conducted, HPLC analyses were performed to rule out IBU degradation using a mixture of IBU and Neusilin in a 1:1 ratio. The feeding rate and the screw speed were set at their minimal proposed levels, i.e., 100 g/h and 20 RPM, respectively. This approach was considered a worst-case scenario due to the longest possible residence time. The longer a processed mixture spends in a machine, the more thermal and mechanical stress is applied, theoretically causing higher degradation rates or evaporation during the HMI of the processed active compound. The obtained results indicated that no IBU degradation or evaporation occurred under tested conditions. The quantity of IBU of the investigated samples was practically the same as the nominal value of 200 mg (99.4% ± 0.39%). Moreover, no additional peaks were observed on the chromatograms that would indicate IBU decadence. These results agree with the observation described by Krupa et al. and indicated that IBU is stable and present in the sample. Probably, the interaction between a carboxyl group of IBU and OH group from the surface of Neusilin prevents the evaporation of IBU during HME [18]. 

### 2.1. Thermal Analysis of Hot Melt Impregnated Samples

**Figure 1:**
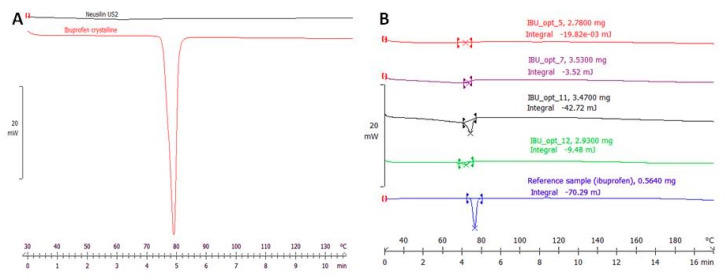
(**A**) Thermal analysis of crystalline ibuprofen (IBU) and amorphous Neusilin US2. (**B**) Differential scanning calorimetry (DSC) thermograms of samples obtained from the hot melt impregnation (HMI) process as examples.

To quantify the IBU amorphization ratio, the crystallinity degree of the sample was measured by DSC. Generally, the reduction of crystallinity in the sample was proportional to the increase in amorphous IBU. Figure 1 presents the thermograms of the tested samples containing amorphous magnesium aluminometasilicate and crystalline IBU. 

The experimentally determined heat of fusion (enthalpy of melting) of IBU was 123.45 mJ/mg, which only slightly differs from 128 mJ/mg of literature data [30]. In Table S2, the experimental heat of fusion values of the crystalline IBU samples are presented. Figure S3 presents exemplary DSC thermograms. 

Knowing the melting enthalpy and the exact weight of the analyzed sample allowed for the determination of the crystallinity of the sample. This principle was utilized to determine residual crystallinity in the samples after HMI. Initially, only IBU showed a crystalline structure in the mixtures, indicating that any thermal effect measured during the analysis came from IBU. The amount of heat measured during melting was proportional to the mass of crystalline IBU in the sample. The relationship between the theoretical and measured heat transfer values determined the crystallinity percentage of IBU in the sample. This method was used to evaluate the percent of residual crystallinity of IBU in the prepared extrudates after HMI (example DSC thermograms are presented in Figure 1B).

### 2.2. DoE of the HMI Process

The HMI experiments were conducted according to the generated experimental plan based on a central composite design, with the main aim being to explore the effects of each input factor and find optimal combinations to assure the most efficient amorphization rate. Taking into account the theoretical assumptions, three input factors were selected as the most probable factors influencing IBU amorphization in the Neusilin matrix, namely, feeding rate, screw speed, and IBU content, which were tested in proposed ranges. Other parameters, such as temperature profiles (70/110/170/130 °C), screw dimensions (20:1), cooling conditions (freely cooled to room temperature), and environmental conditions (temperature below 25 °C and humidity below 60%), were kept constant. As an output variable, the crystallinity levels of IBU were measured using DSC. All prepared compositions after extrusion were analyzed. Endothermic effects were visible at around 75 °C, thereby indicating the presence of crystalline IBU in the extrudate, with a lack of observed thermal effects for all compositions identifying an amorphous nature of the tested samples. From a manufacturing point of view, this analysis also confirmed the appropriate process parameters. All the prepared extrudates were white to off-white, free-flowing powders, therefore, no additional milling/grinding step was applied. The obtained results allowed statistical analyses to be performed. Table 1 shows the determined crystallinity levels of the IBU in each extrudate, demonstrating that an increased screw speed caused an increased crystalline IBU to remain. A reduction of the crystalline fraction in the samples was promoted by elongation of the residual time. 

The next step of the investigation concerned the assessment of the established DoE model. In order to evaluate the model’s significance, summary statistics and the ANOVA analysis were performed. The first bar in the summary of the fit plot was the R^2^ value, which is the fraction of the variation of the response as explained by the model. The obtained R^2^ was very high (0.98) and indicated a good significance of the proposed model. The second assessment bar was Q^2^, which shows the ability to generalize to unseen data. Q^2^ is also known as the model’s predictive power. The calculated Q^2^ was close to 0.80, indicating that the proposed model had a significant predictive ability. The other evaluated model fit plot was the model validity, which gives information about the model error in terms of pure error. The obtained value was 0.28, meaning that the obtained model met the criteria (>0.25), therefore, the model had no lack-of-fit. The last assessment parameter was reproducibility, which is the variation of the response under the same conditions as the total variation of the response. The computational reproducibility was close to 1 (exact value 0.99), indicating a perfect robustness of the process [28]. 

The ANOVA analysis was performed to determine the influence that the input variables had on the output variable in a regression study. This analysis explains the probability of observed differences between the experimental runs for the selected factors. Details of the ANOVA analysis are presented in Table 2. As expected, the obtained model was statistically insignificant at p = 0.05, whereas the F-test indicated that the linear model adequately explained the response. 

**Figure 2:**
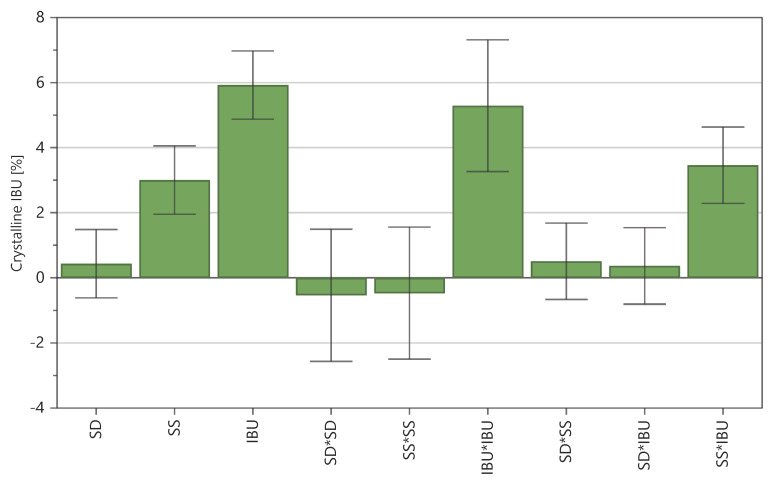
The obtained coefficient values of each input factor. IBU: IBU content in the sample; SD: Feeding rate; SS: Screw speed.

The influences of the individual factors on the response were then evaluated. Figure 2 presents the coefficient values of each input factor, with a positive value indicating that an increase of a factor occurred alongside an increased response value. 

**Figure 3:**
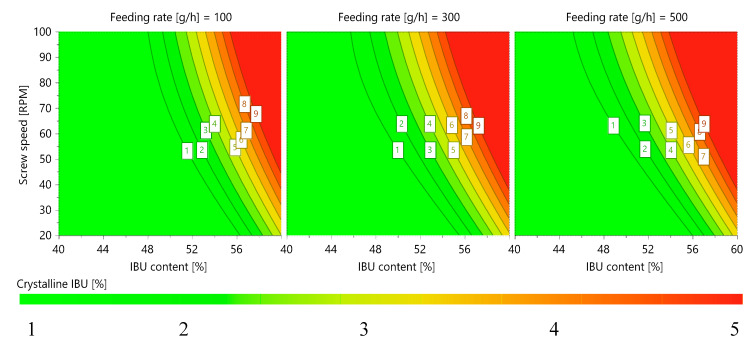
Four-dimensional (4D) contour chart of IBU crystallinity in the samples after the impregnation process.

All three input factors were positive, however, the feeding rate (SD) was not statistically significant, as clearly illustrated by confidence intervals which crossed the x-axis; therefore, the coefficient was not considered to be important. This finding also confirmed a graphical representation of the response surface, which presented the multidimensional space as a combination of input factors and the response of the experiment. To show crystallinity results as a function of all three input factors, 4D charts were prepared. Figure 3 presents the crystallinity levels as functions of the feeding rate, screw speed, and IBU content, showing that the crystallinity levels of the tested samples depended only on the combination of the screw speed and IBU content. For further studies, the model was modified and the feeding rate was excluded for the regression equation. 

Upon interpretation of the adjusted model, the following conclusions can be drawn. The most important factor that influences the amorphization effectiveness is the IBU content, which determines the mass ratio of API to the carrier. Generally, the impregnation of IBU is less effective when there is less from the Neusilin carrier. The screw speed strictly corresponds to time, and this relationship is needed for a material to go through the equipment. A slower rotation of the screw means a longer exposure to the heat inside the extruder and thus more effective impregnation. The feeding rate is generally responsible for the overall process yield; therefore, it can be understood as a product manufactured over time. This parameter has an impact on the residence time and degree of fill inside the barrel, and for many twin-screw processes, the feeding rate is crucial from a technological point-of-view. However, under the test conditions, the feeding rate range had a negligible impact on the amorphization. The obtained results indicated that the feeding rate did not influence any thermodynamic transformation that occurred during amorphization of IBU. Consequently, this parameter was excluded from the proposed model.

The regression equation after adjustment of the model is presented as Equation (1). This regression model allowed the prediction of crystallinity levels of IBU[X] from the proposed HMI process as a function of the IBU content (expressed as mass percentage in the sample) and screw speed (expressed as revolutions per minute, RPM). It should be emphasized that the R^2^ for this model was 0.98, meaning that 98% of the response variance was explained by the model.

X= −0.357 SS − 4.699 IBU + 0.048 IBU^2^ + 0.00864 SS∙IBU + 112.34 (1)

where X is the crystallinity level (mass percentage) of IBU in the sample after the process; IBU is the IBU content in the sample, expressed as mass percentage; SS is the screw speed, expressed as revolutions per minute.

**Figure 4:**
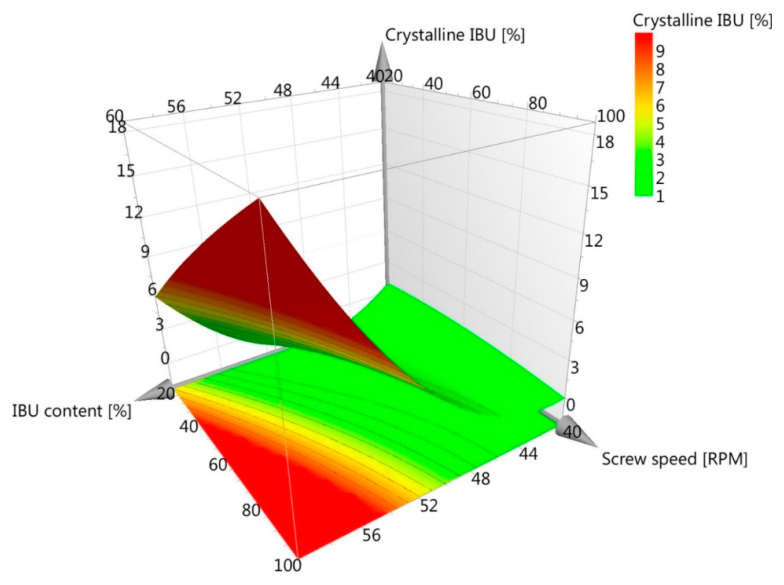
Surface response plot for the adjusted model.

The presented equation is a mathematical representation of the model, where the predicted crystallinity can be calculated by inputting values of the impregnated IBU for any combination of input factors within the tested range. Graphically, results were presented as a three-dimensional response surface plot (Figure 4), showing that increases in screw speed and IBU content in the model led to higher residual crystallinity in the impregnated material. By controlling these two parameters, the required amorphization of IBU during HMI was ensured.

**Figure 5:**
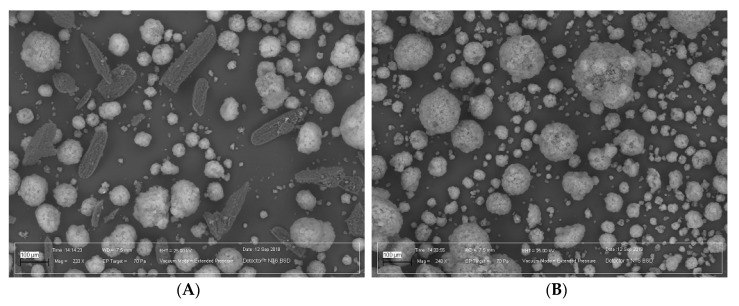
SEM images of (**A**) the unprocessed physical mixture (IBU 50%, Neusilin 50%) and (**B**) the HMI extrudate. Magnification: 144×.

To verify the correctness of the obtained model, a test batch containing 50% IBU and 50% Neusilin was manufactured by HMI, and the obtained extrudates were thoroughly examined using SEM, XRPD, and DSC. The SEM images of the test composition before and after HMI are shown in Figure 5. 

**Figure 6:**
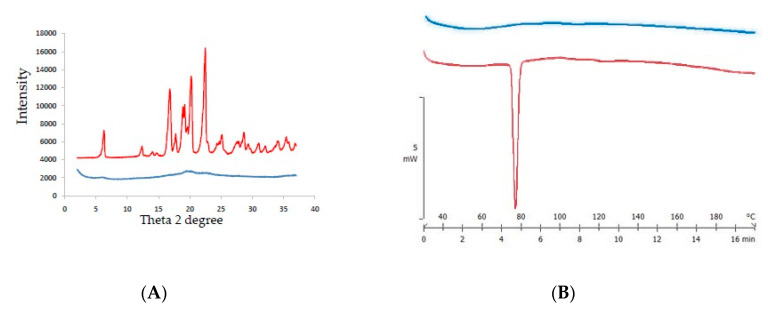
(**A**) XRPD diffractogram and (**B**) DSC thermograms of the physical mixture (IBU 50%, Neusilin 50% (red)) and the HMI extrudate (IBU 50%, Neusilin 50% (blue)).

Figure 5 shows that IBU was completely incorporated into the internal Neusilin pores, therefore, after HMI, only Neusilin particles were observed. This finding also confirmed the X-ray and DSC analysis results of the prepared test batch, proving that IBU was present in the samples as an amorphous structure after HMI (Figure 6). 

The XRPD diffractogram showed the typical signal for IBU crystallinity in a physical mixture, which was contrary to the HMI extrudate sample. A DSC analysis confirmed these results, as endothermic effects visible at around 75 °C indicated the presence of crystalline IBU. In the analyzed impregnate, no endothermic effects were observed, thereby confirming the amorphous nature of the tested samples. Taking into account the assumptions of the proposed model, a fully amorphous material after HMI was obtained at the given conditions (feeding rate 300 g/h, screw speed 50 RPM, IBU content 50%).

**Figure 7:**
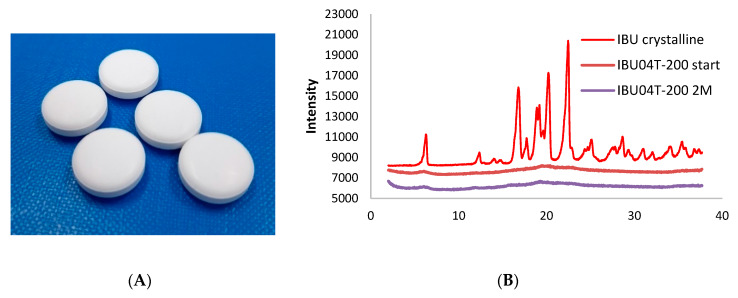
(**A**) Tablets containing IBU–Neusilin extrudates, (**B**) XRPD diffractograms of IBU tablets at different time points, stored in 40 °C/75% RH in PVC blisters after two months (violet line).

The final step of our investigation was the verification that the obtained extrudate with an amorphous IBU can be used for the tableting process. Moreover, the comparison of the dissolution profile as well as assessment of the thermodynamic stability of the obtained tablets with amorphous IBU and Neusilin were performed. Prepared tablets (IBU04T-200) were manufactured from previously impregnated material and they are presented in Figure 7A.

The XRPD diffractogram indicated that an amorphous IBU impregnated into Neusilin structures is stable during the tableting process. The achieved results suggest that during tableting, we do not observe destruction of the amorphous IBU–Neusilin structure, which could cause recrystallization of IBU. Furthermore, the preliminary physical stability tests demonstrated that the target formulation causes no recrystallization within two months of accelerated stability study, further time points are ongoing. Currently, investigating the stability is further continued.

**Figure 8:**
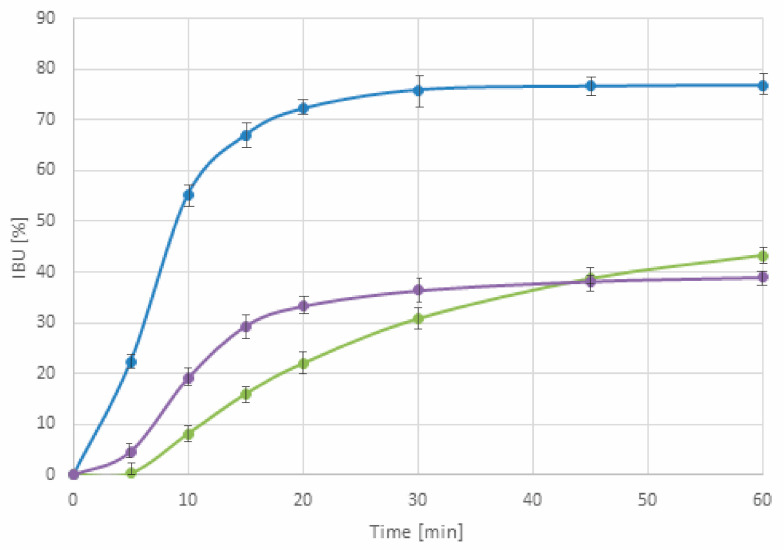
Dissolution profiles for tablets containing IBU–Neusilin extrudates (blue line) and commercially available product containing ibuprofen tablets (green line) and soft gelatin capsules in 0.1 M hydrochloric acid, 900 mL, 50 RPM, paddle apparatus.

Figure 8 showed that amorphization enhances the dissolution rate of IBU. Tablets containing IBU–Neusilin extrudates exhibited a significantly higher dissolution rate in comparison to commercially available products containing IBU tablets and soft gelatin capsules. The percentage of the released IBU in 30 and 60 min were two- to three-times higher. It is worth emphasizing that the faster an active substance is released, the quicker the therapeutic effect is, which is a preferable property for painkiller drugs.

## 3. Materials and Methods 

### 3.1. Chemicals

IBU was purchased from Strides Shasun Ltd. (Bangalore, India) and magnesium aluminometasilicate was bought from Fuji Chemical Industries Co., Ltd. (Toyama, Japan) under the branded name Neusilin, grade US2. Florite PS-200 were purchased from Tomita Pharmaceuticals Ltd. (Tokyo, Japan) and Syloid XDP 3150 were kindly donated by W.R. Grace (Columbia, CA, USA). Croscarmellose sodium was donated by JRS Pharma (Rosenberg, Germany). Magnesium stearate was purchased from FACI s.p.a. (Carasco, Italy). Acetonitrile-grade liquid chromatography was obtained from Merck (Darmstadt, Germany). Aluminum liding foil were purchased from Amcor Flexibles Kreuzlingen AG (Kreuzlingen, Swizerland). PVC of 250 um foil was purchased from MKF-Ergis (Wąbrzeźno, Poland). The hydrochloric acid analytical grade was obtained from POCH (Gliwice, Poland). Commercially available drugs with IBU (200 mg) in both tablets and soft gel capsules were bought from the local drugstore. 

### 3.2. Preparation of IBU Physical Mixtures

After weighing, the materials were sieved through a 1.0 mm sieve. The IBU–Neusilin mixtures were then blended in a 2 L stainless steel bin container and rotated at 12 RPM for 10 min in a rotary bin blender from Pharmatech, Multiblend MB015 (Coleshill, United Kingdom). Three different compositions were examined: IBU–Neusilin 40%:60%, IBU–Neusilin 50%:50%, and IBU–Neusilin 60%:40%.

### 3.3. Hot Melt Impregnation Process

**Figure 9:**
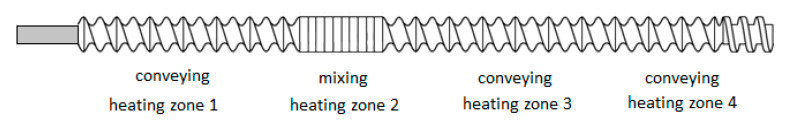
Screw configuration used in the HMI process.

The hot melt impregnation process was conducted with a twin screw (co-rotating) extruder ThreeTec ZE 18 (Seon, Switzerland) with an L/D ratio of 20:1, including four heating zones. The optimal temperature profile of the process was chosen experimentally and described by our previous work [5]. The final temperature profile was set as 70/110/170/130 °C and was applied for all of the following impregnation runs. The screw configuration was kept constant and proposed as shown in Figure 9. Only a short mixing section was applied to the conveying elements to minimize the shear stress during the process, as shear force can damage the mesoporous Neusilin structure. Element mixing was performed to evenly distribute the melted IBU within the rest of the material, thereby maximizing the contact area and intensifying the absorptive effect. The feeding rate, screw speed, and IBU content were chosen as parameters to be optimized in the ranges of 100 to 500 g/h, 20 to 100 RPM, and 40% to 60%, respectively. The experimental matrix of the design experimental plan is presented in the statistical analysis section.

### 3.4. DSC Analysis.

The thermal properties of IBU, Neusilin, and the examined physical mixtures and extrudates after HMI were investigated using the Mettler-Toledo Star 1 (Greifensee, Switzerland) apparatus. Samples ranging from 0.1 to 2 mg in mass were placed in a standard aluminum pan with a pierced lid and heated at 10 °C/min from 30 to 200 °C. Before the start of work each day, the DSC instrument was calibrated with respect to Zn and In. All experiments were carried out under a nitrogen flow of 30 mL/min. The observed thermal effects were quantified and recalculated to the residual IBU content.

### 3.5. XRPD

X-ray analyses of samples of physical mixtures and extruded materials were conducted using a powder diffractometer from Rigaku Miniflex (Tokyo, Japan). The XRPD apparatus was used to count the detected intensity as a function of the angle between the incident and the diffracted beam. A Cu anode at 30 kV and 15 mA with a Kβ foil filter, a variable divergence slit, a fixed scattering slit at 4.2 mm, a fixed receiving slit at 0.3 mm, a Soller slit at 2.5°, and a NaI scintillator as a detector were used during this study. After the sample was placed on a silicon holder, the scanning program was applied with the parameters of 2–38° 2θ, with a sampling width of 0.02°, and a scan speed of 1.00°/min.

### 3.6. SEM

The surface morphologies of the physical mixtures and extruded materials were visualized using the Carl Zeiss EVO/LS25 Scanning Electron Microscope (Oberkochen, Germany) equipment with a backscattered electron (BSE) detector. Before analysis, the samples were mounted on an aluminum stage using an adhesive carbon tape. The experiments were carried out in a variable pressure mode in a nitrogen gas environment. The gas pressure in the chamber ranged from 60 to 100 Pa at an accelerating voltage of 20 kV. This method allows for the analysis of non-conductive samples, since it reduces the sample surface charging effect [31].

### 3.7. Experimental Plan and Statistical Analysis

For DoE purposes and statistical analyses, the MODDE Pro 12 (Malmo, Sweden) software was used and a face-centered composite design was chosen. The experimental plan, with three input factors at three levels and three repetitions at central points, was established using algorithms implemented into the MODDE Pro 12 software. The total number of runs was 17. Optimization of the hot melt impregnation process to develop an amorphous IBU solid dispersion was the aim of the design. The tested factors were feeding rate, screw speed, and IBU content, with the temperature profile and screw design kept at constant. As a response of the design, the content of crystalline IBU was measured. The run sequence was randomized according to the scheme presented in Table 3. The graphically experimental matrix is shown in Figure S4.

### 3.8. HPLC Analysis

The HPLC analysis was carried out using the Waters Alliance 2695 Separations HPLC System (Waters Corporation, Milford, USA) equipped with an ACE C_18_-HPLC column (150 × 4.6 mm × 5 μm column, ACE^®^, Aberdeen, Scotland). Before injection, sample solutions were filtered through a 0.45 μm GHP syringe filter (PALL Corporation, Port Washington, New York, NY, USA). A binary mobile phase consisting of 25% aqueous solution (0.1%) and 75% acetonitrile was used. The isocratic flow was set at 1 mL/min and the injection volume was 10 μL. The column oven and autosampler temperature were constant at 25 °C. The detection of IBU was performed at 273 nm.

### 3.9. Dissolution Study

An in vitro dissolution study was conducted on IBU products 200 mg in strength. The drug release was carried out in 900 mL of 0.1 N HCl, paddle apparatus (paddle speed 50 RPM). Dissolution bath and vessels were equilibrated to 37 ± 0.5 °C. During the test no sink conditions were fulfilled. At predetermined time points, samples were collected (5, 10, 15, 20, 30, 45, and 60 min). The test was performed on the DISTEK Evolution 6100 (Distek, North Brunswick, NJ, USA). Collected samples were analyzed using UV–Vis spectroscopy (Agilent 8453 spectrophotometer, Agilent Technologies, Palo Alto, CA, USA). Samples were collected automatically from dissolution vessels by the peristaltic pump Agilent 8VS (Agilent Technologies, Palo Alto, CA, USA) and analyzed at 221 nm wavelength. Analyses were performed for six formulations of each type and the standard deviation of obtained results was below 5%.

### 3.10. Tablet Compression

Tablet compression has been conducted on a laboratory rotary tablet press Killian IMA Pressima (Killian, Cologne, Germany). Formulation IBU04 (consisting of 96% impregnated Neusilin, 3% croscarmellose sodium, and 1% magnesium stearate) was compressed into Ø12 mm biconvex round tablets, each tablet contained 200 mg of ibuprofen in an inorganic carrier. The tableting blend was fed through the feeder with paddles. The rotation speed of the turret was set at 25 RPM. A pre-compression force equaled 0.5 kN, and the main compression force was set at 5 kN.

### 3.11. Tablets Blistering

Prepared tablets were packed in PVC/Al blisters. The O.M.A.R. Fantasy Plus Blistering machine (O.M.A.R. Milan, Italy) was utilized. The sealing temperature was set at 135 °C, the sealing time was equal to 1.5 s. For blistering, previously formed PVC 250 um thickness blisters were used. Blister forming was conducted at 125 °C.

### 3.12. Stability Testing

The accelerated stability study was conducted in a validated climatic chamber KK750 (POL-EKO Aparatura (Wodzisław Śląski, Poland). A climatic chamber was validated to conditions of 40 °C and 75% RH. Tablets packed in PVC/Al blisters were placed in a chamber and the influence of storage time were tested.

## 4. Conclusions

The discussed method provides a fresh new look regarding the optimization of HMI in terms of amorphization efficiency. The developed model allowed the prediction of processing parameters, which should be applied in order to manufacture completely amorphous solid-phase dispersions. Among the target process parameters, the IBU content and screw speed significantly influenced the crystallinity level of IBU, whereas the feeding rate did not have an impact on IBU amorphization. The accuracy of the proposed model confirmed verification of the test batch, which was subjected to SEM, XRPD, and DSC analysis. Taking into account the similarities between amorphous API and the matrix substance complex formations, which are based on physical mechanisms such as pore size, we believe that the proposed approach could be simply transferred to optimize the HMI process for other mixtures. This assumption supports reports of other chemically diverse substances such as ketoprofen, indomethacin, naproxen, or progesterone [11,18], which have similar stabilities as the amorphous forms of the Neusilin-complex formations after HMI. This topic will be tested in our future work.

## Figures and Tables

**Table 1:** Experimentally determined crystallinity levels of IBU and HMI conditions of investigated extrudates.

Exp. No.	Input Factors			Output Factors
	Feeding Rate [g/h]	Screw Speed [RPM]	IBU Content in the Sample [%]	Crystallinity Level of IBU [%]
1	100	20	40	Not detected
2	500	20	40	0.20
3	100	100	40	Not detected
4	500	100	40	0.11
5	100	20	60	4.71
6	500	20	60	4.25
7	100	100	60	16.43
8	500	100	60	20.11
9	100	60	50	0.82
10	500	60	50	1.59
11	300	20	50	Not detected
12	300	100	50	2.54
13	300	60	40	Not detected
14	300	60	60	14.06
15	300	60	50	0.82
16	300	60	50	1.12
17	300	60	50	1.61

**Table 2:** Statistical significance of factor effects based on the ANOVA analysis.

Crystalline IBU	DF	SS	MS (Variance)	F	p	SD
Total	17	926.510	54.501			
Constant	1	274.968	274.968			
Total corrected	16	651.542	40.721			6.381
Regression	9	637.748	70.861	35.958	0.000	8.418
Residual	7	13.7945	1.9707			1.404

**Table 3:** Experimental design for the proposed central composite design plan.

Exp. No.	Run Order	Real Values of Input Variables		
		Feeding Rate [g/h]	Screw Speed [RPM]	IBU Content [%]
1	10	100	20	40
2	17	500	20	40
3	9	100	100	40
4	6	500	100	40
5	7	100	20	60
6	8	500	20	60
7	13	100	100	60
8	3	500	100	60
9	16	100	60	50
10	11	500	60	50
11	1	300	20	50
12	2	300	100	50
13	4	300	60	40
14	12	300	60	60
15	14	300	60	50
16	15	300	60	50
17	5	300	60	50

## Supplementary Materials

Supplementary materials can be found at https://www.mdpi.com/1422-0067/21/11/4032/s1, Table S1: Comparison of Neusilin US2 Florite PS-200 Syloid XDP 3150 properties; Table S2. Determination of ibuprofen heat of fusion (melting enthalpy) using DSC; Figure S1. SEM image of Neusilin US2: (a) Florite PS-200, (b) Syloid XDp 3150; Figure S2. XRPD diffractograms of extrudates based on different carriers; Figure S3. Thermal effects of different amounts of crystalline ibuprofen; Figure S4. Experimental matrix presented as a multidimensional space.

## Abbreviations

HME	Hot melt extrusion
API	Active pharmaceutical ingredient
HMI	Hot melt impregnation
DoE	Design of experiments
RMS	Response surface methodology
IBU	Ibuprofen
DSC	Differential scanning calorimetry
HPLC	High-performance liquid chromatography
SEM	Scanning electron micrograph
XRPD	X-ray powder diffraction
