# Supplementary material for: A Novel Approach to Optimize Hot Melt Impregnation in Terms of Amorphization Efficiency
Source: Int J Mol Sci. 2020 Jun 4;21(11):4032. doi: 10.3390/ijms21114032 (PMC7312772; doi:10.3390/ijms21114032)
Supplement: Supplementary file 1 [file ijms-21-04032-s001.pdf]

Supplementary materials

A novel approach to optimize hot melt impregnation in terms of amorphization efficiency

Kamil Garbera[1], Krzesimir Ciura[2], Wiesław Sawicki[2*]

[1] Tarchomin Pharmaceutical Works "Polfa" S.A., Formulation Department, Fleminga 2, 03-176 Warszawa, Poland; kamil.garbera@gmail.com

[2] Medical University of Gdańsk, Department of Physical Chemistry, Hallera 107, 80-416 Gdańsk, Poland;krzesimir.ciura@gumed.edu.pl

* Correspondence: Wiesław Sawicki Medical University of Gdańsk, Department of Physical Chemistry, Hallera 107, 80-416 Gdansk, Poland e-mail: wsawicki@gumed.edu.pl

Table 1. Comparison of Neusilin US2 Florite PS-200 Syloid XDP 3150 properties*.

	Neusilin US2	Florite PS-200	Syloid XDP 3150
**Chemical name**	magnesium aluminosilicate	Calcium silicate	Silicon dioxide
**Appearance**	White granules	White fine granulate	White free flowing powder
**Oil absorption [ml/g]**	2,7-3,4	3,7	3,0
**Bulk densitiy [g/cm3]**	0,13-0,18	0,07	0,275
**Average particle size [um]**	106	150	150
**pH as 1 % aqueous slurry**	6-8	8,4-11,2	4,0 – 7,0
**Specific surface area [m2/g]**	420	130	320
**Average pore size [nm]**	15	200	10

* based on sellers' documents.

Table 2. Determination of IBU heat of fusion (melting enthalpy) using DSC.

Sample number	Sample weight	Heat measured during melting	Heat of fusion of the sample	Average heat of fusion (n = 6)
	[mg]	[mJ]	[mJ/mg]	[mJ/mg]
1	0.127	14.73	115.98	
2	0.132	15.47	117.20	
3	0.564	70.67	125.30	123.45
4	0.539	67.19	124.66	
5	1.004	130.9	130.38	
6	1.021	129.85	127.18	

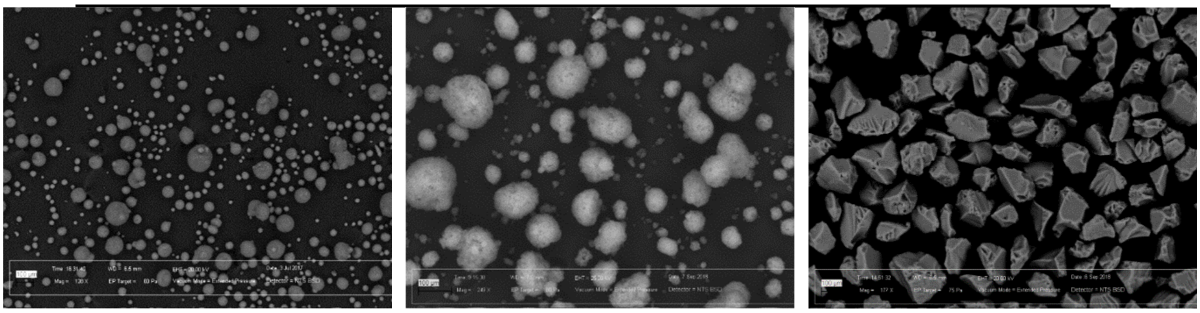

(**A**) (**B**) (**C**)

Figure S1. SEM image Neusilin US2 (**A**), Florite PS-200 (**B**), Syloid XDp 3150 (**C**).

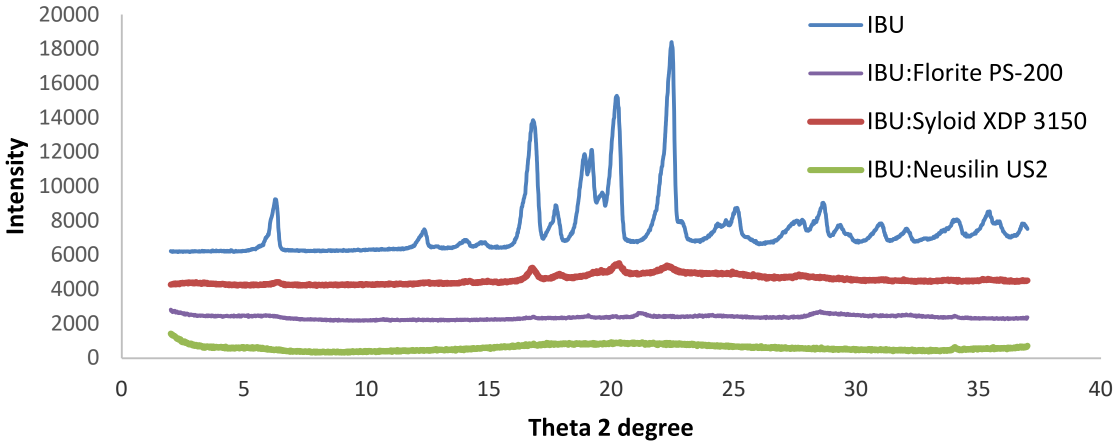

Figure S2. XRPD difractograms of extrudaes based on different carriers.

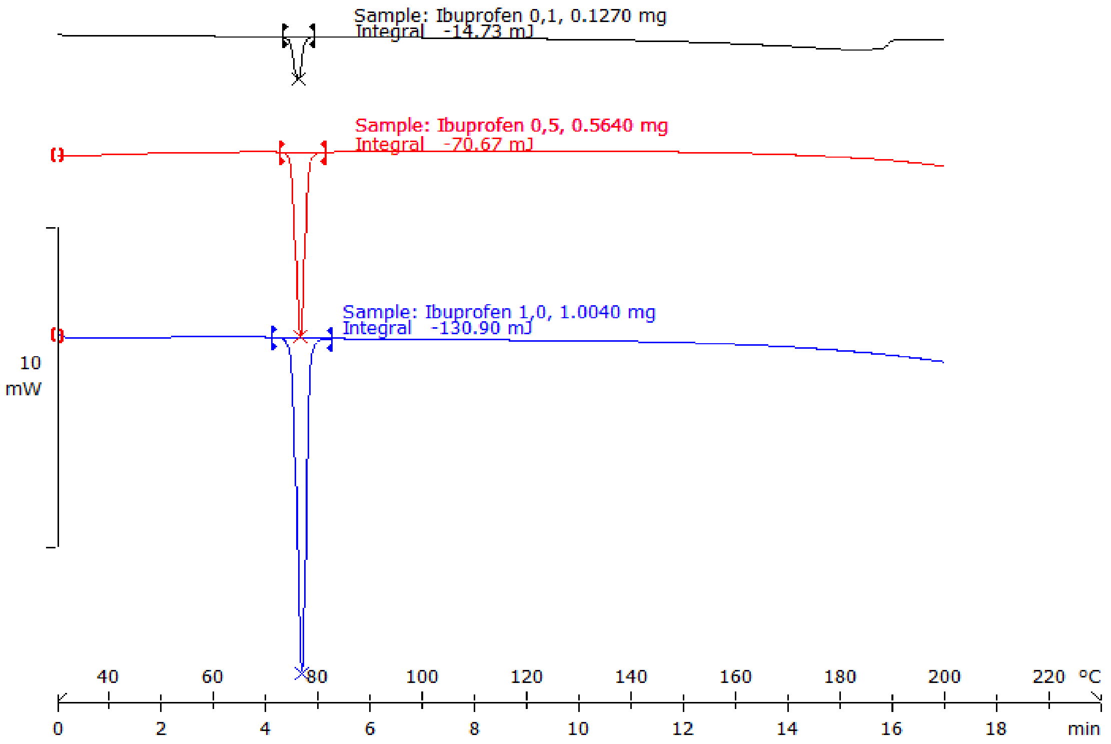

Figure S3. Thermal effects of different amounts of crystalline IBU.

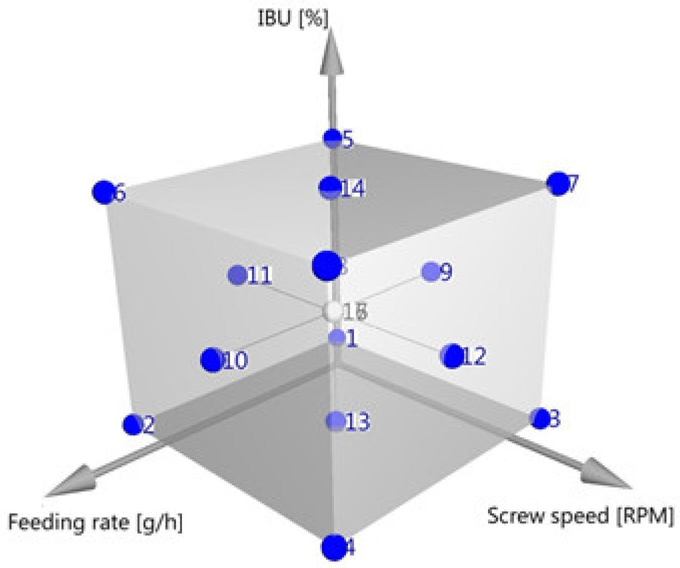

Figure S4. The experimental matrix.
